# The Effects of Circadian Rhythms and Exercise Preconditioning on Cardiac Troponin T Levels Following Graded Exercise

**DOI:** 10.1002/ejsc.12294

**Published:** 2025-04-06

**Authors:** Jinlei Nie, Ruoyu Zhang, Haifeng Zhang, Qingde Shi, Keith George, Zhaowei Kong

**Affiliations:** ^1^ Faculty of Health Sciences and Sports Macao Polytechnic University Macau S.A.R China; ^2^ Department of Rehabilitation Renmin Hospital of Wuhan University Wuhan China; ^3^ School of Rehabilitation Sciences and Engineering University of Health and Rehabilitation Sciences Qingdao China; ^4^ Research Institute for Sport and Exercise Sciences Liverpool John Moores University Liverpool UK; ^5^ Faculty of Education University of Macau Macau S.A.R China

**Keywords:** cardiac biomarker, circadian rhythms, exercise preconditioning, graded exercise test

## Abstract

This study explored the impact of circadian rhythms on the circulating cardiac troponin T (cTnT) response to a graded exercise test (GXT) and examined whether an initial GXT influenced the cTnT response to a subsequent GXT performed 7–9 days later. Twenty‐one healthy young males (age: 20.6 ± 2.2 years, body mass index: 22.2 ± 2.6 kg/m^2^, V̇O_2max_: 31.8 ± 8.7 mL.kg^−1^.min^−1^) participated in three trials: an initial GXT (GXT1), a resting control trial (CON) and a second GXT (GXT2), separated by at least 72 h. The serum cTnT levels were measured pre‐exercise, 4 h post‐exercise or during the control. In GXT1, the cTnT levels did not show significant changes (median [range], pre: 3.80 [3.00–10.59] ng.L^−1^, post: 4.22 [3.00–9.08] ng.L^−1^, *p* > 0.05). During CON, the cTnT levels decreased significantly from morning to early afternoon (3.52 [3.00–10.84] vs. 3.00 [3.00–7.57] ng.L^−1^, *p* < 0.05), reflecting a circadian rhythm. Interestingly, GXT1 appeared to prevent this circadian decline. Furthermore, in GXT2, the cTnT levels significantly decreased post‐exercise (4.13 [3.00–15.48] vs. 3.24 [3.00–12.96] ng.L^−1^, *p* < 0.05), suggesting a possible “late exercise preconditioning” effect from GXT1. These findings suggest that GXT can interact with circadian rhythms, altering cTnT dynamics, and that prior exercise may induce prolonged cardioprotective effects. This study highlights the importance of accounting for circadian variability and late preconditioning effects in future research on exercise‐induced cTnT release.

**Summary**

- Graded exercise testing (GXT) prevents the natural circadian decline in cardiac troponin T (cTnT) levels, indicating a possible interaction between exercise and circadian rhythms in young males.
- A second GXT, conducted 7−9 days after an initial GXT, reduces cTnT levels, suggesting a potential late exercise preconditioning effect.

## 1 Introduction

An elevation in circulating cardiac troponin (cTn), specifically cardiac troponin T (cTnT) and I (cTnI), is a gold standard biomarker response that reflects myocardial damage (Byrne et al. 2024). In addition, high resting levels of cTn are associated with a poor prognosis in patients with coronary artery disease as well as in healthy individuals (Farmakis et al. 2020; McEvoy et al. 2016). Prolonged strenuous exercise has also been shown to result in elevated cTn levels in healthy individuals (Aengevaeren et al. 2021). Although this phenomenon has typically been viewed as a benign physiological response (Aengevaeren et al. 2021), recent studies involving 30−55 km walking tests and 91 km mountain bike races have shown independent links between exercise‐induced cTn elevation, adverse cardiovascular events and obstructive coronary artery disease (Aengevaeren et al. 2019; Kleiven et al. 2020; Skadberg et al. 2017). These findings suggest that post‐exercise cTn measurement could serve some diagnostic or prognostic role (Nie 2018). However, the current protocols involving prolonged and strenuous exercise are impractical for clinical application. Evaluation of shorter, standardised exercise tests, which are common in clinical practice, and their cTnT response would be helpful to determine any diagnostic potential.

A graded exercise test (GXT) is a widely used and standardised clinical test for assessing cardiovascular function and health. Research indicates that a GXT can lead to an increase in circulating cTn levels in individuals with various health conditions (Bjørkavoll‐Bergseth et al. 2021; Bjorkavoll‐Bergseth et al. 2024; Samaha et al. 2019), suggesting its potential as a screening tool for detecting exercise‐induced cTn elevations in the general population. However, the total exercise exposure of a GXT is much lower than that of prolonged and/or strenuous exercise, and the reported elevations in cTn levels are smaller. In such scenarios, the circadian variation in cTnT, which can exceed 20% (Klinkenberg et al. 2014), may complicate the potential clinical value of cTnT responses to a GXT. Hence, clarification of the relationship between post‐GXT cTnT changes and circadian variation may help illuminate the diagnostic/prognostic value of a cTnT response to GXT.

To further enhance our understanding of cTn levels after GXT, it is also crucial to explore the concept of “exercise preconditioning” and the potential that prior exercise could blunt the cTn response to subsequent activity. Increasing evidence, primarily from animal studies, indicates that a single exercise bout provides immediate cardioprotection, such as a reduced infarct size, known as exercise preconditioning (Thijssen et al. 2018, 2022). This preconditioning effect typically exhibits a biphasic cardioprotective response: “early protection” occurs immediately after one session of exercise and lasts for about 4 h, whereas “late protection” reappears 36 h after exercise and can last for several days (Thijssen et al. 2018). We previously found in humans that the significant elevation in cTnT levels after a first exercise bout was markedly reduced after a second, identical bout separated by either 4 or 48 h of recovery (Nie, George, Tong, Tian, et al. 2011; Zhang et al. 2019). This suggests that exercise preconditioning during the “early or late protection” phase may blunt or eliminate the cTnT response to acute exercise. Although animal studies have demonstrated that exercise preconditioning can induce cardioprotection for up to 9 days (Thijssen et al. 2018), there are limited data in humans on whether this protection, which influences the post‐exercise cTn response, persists for up to 1 week. Thus, it is important to investigate whether post‐GXT cTn levels are influenced by exercise performed up to 1 week earlier to determine the optimal timing for testing to accurately assess post‐GXT cTn levels.

Consequently, this study aims to examine (1) whether a single GXT modulates the natural circadian variation in circulating cTnT levels and (2) whether an initial GXT influences the cTnT response to a subsequent GXT performed 7–9 days later. To our knowledge, this is the first study to investigate both the interaction between GXT and circadian fluctuations in cTnT levels and the potential late‐phase cardioprotective effects of prior exercise in a human cohort. Unlike previous studies that have primarily focused on prolonged exercise, we have uniquely examined the effects of a standardised, clinically relevant GXT protocol, thereby improving its translational potential for cardiovascular risk assessment. Given that young individuals with low cardiovascular risk may benefit more from cTn‐based cardiovascular stratification tools compared with existing screening methods (Farmakis et al. 2020), we specifically recruited healthy young males. We tested the following hypotheses: (1) cTnT levels decline during the control condition due to circadian variation, but this decline is attenuated following GXT, and (2) an initial GXT attenuates the cTnT response to a subsequent GXT. Considering the growing interest in the diagnostic and prognostic value of exercise‐induced cTnT (Aengevaeren et al. 2019; Kleiven et al. 2020; Skadberg et al. 2017), these findings may provide novel insights into optimising exercise testing protocols for cardiovascular screening and risk stratification.

## 2 Materials and Methods

### 2.1 Participants

The inclusion criteria for participants were as follows: (1) age between 18 and 25 years; (2) body mass index (BMI) < 25 kg.m^−2^, based on the World Health Organization (WHO 2000) obesity cutoff for Asian adults; (3) no regular physical activity or structured exercise training (≤ 2 sessions per week of low‐intensity exercise) and (4) no personal history of hormonal, orthopaedic or cardiovascular diseases, diabetes, hyperlipidaemia, hypertension or use of prescribed medication. The exclusion criteria included the following: (1) any chronic medical condition that could interfere with exercise testing; (2) a history of smoking or alcohol abuse; (3) irregular sleep patterns or shift work within the past 3 months; (4) significant weight change (± 2 kg) in the past 3 months and (5) a family history of premature cardiovascular disease.

A total of 58 volunteers were recruited from local universities through advertisements. Of these, 26 males were deemed eligible based on the inclusion and exclusion criteria. Following recruitment, five eligible participants withdrew from the study for personal reasons, such as schedule conflicts and illness. Consequently, a final cohort of 21 participants (mean ± standard deviation [SD]: age 20.6 ± 2.2 years, height 174.7 ± 7.4 cm, body mass 68.1 ± 13.6 kg and BMI 22.2 ± 2.6 kg.m^−2^) completed the study and were included in the analysis. All participants were fully informed about the nature of the study and provided written informed consent. The study protocol was approved by the regional ethics committee for research involving human subjects.

### 2.2 Experimental Design and Procedures

**FIGURE 1:**
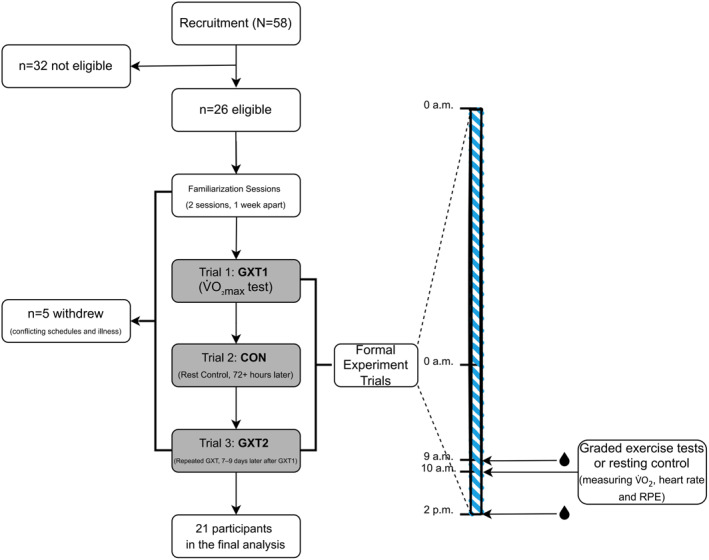
Recruitment, retention and the experimental design. **GXT1**, the first trial, a graded exercise test to determine V̇O_2max_; **CON**, the second trial, resting control; **GXT2**, the third trial, the same graded exercise test as GXT1; 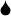, collection of blood samples to determine the levels of cardiac troponin T and/or hormones; 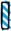 wearing an ActiGraph accelerometer.

Figure 1 details recruitment, retention and the experimental design. After taking anthropometric measurements, two initial familiarisation sessions, spaced 1 week apart, were held in the laboratory to acclimate the participants to a cycling GXT. One week after the final familiarisation session, the subjects commenced the formal trials, which consisted of three separate sessions. (1) GXT1—in this trial, the participants performed a GXT to determine their V̇O_2max_ using a cycling ergometer to assess their maximal aerobic capacity. (2) Control (CON)—in this trial, the participants rested on a stationary bike for the same duration as the exercise trial. The aim was to account for any diurnal variations in cTnT levels (Klinkenberg et al. 2014). (3) GXT2—the participants performed an identical GXT to GXT1, 7–9 days after GXT1, to determine the potential effects of prior exercise on subsequent cTnT levels. Each trial was separated by at least 72 h to allow for recovery between sessions. The entire experimental protocol, sequentially comprising GXT1, CON and GXT2, took place within 7–9 days.

Heart rate was monitored continuously during GXT1, CON and GTX2 using a portable heart rate monitor (Polar H10, Polar Electro Oy, Kempele, Finland). Immediately after each session (∼10 a.m.), the participants provided their rating of perceived exertion (RPE) using the Borg scale (6–20). Venous blood samples were collected before exercise (pre‐exe, 9 a.m.) and 4 h after exercise (post‐exe, 2 p.m.)—as well as for CON (2 p.m.)—to measure the serum levels of cTnT, testosterone and cortisol (the latter two only before exercise). The timing of the post‐exercise blood samples was based on a previous lab‐based study that demonstrated that serum cTnT concentrations peak 4 h after exercise (Tian et al. 2012). Additionally, 3–4 h post‐GXT is a commonly used observation point to capture the cTnT peak (Liebetrau et al. 2015). On the day before and the day of each trial, the participants’ daily physical activity and sedentary behaviour were monitored using a triaxial accelerometer (ActiGraph wGT3x‐BT, ActiGraph Corporation, Pensacola, FL, USA).

For each session, the participants were provided with two standardised light meals, including water, at 8 a.m. and 12 p.m. The meal regimen was kept consistent across all three sessions. All trials were conducted in a controlled, air‐conditioned laboratory at 20°C with 50% relative humidity. The participants were instructed to maintain their usual daily activities, to avoid strenuous exercise and to refrain from altering their eating and sleeping patterns during the experimental period.

### 2.3 Protocol and Measurements

#### 2.3.1 Graded Exercise Test

For GXT1 and GXT2, the participants performed the same GXT protocol, as described previously (Beltz et al. 2016; Durrer et al. 2017). The participants began pedalling at 25 W for 2 min on an electronically braked cycle ergometer (Lode Excalibur Sport, Lode BV, Groningen, the Netherlands); then the workload was increased by 1 W every 2 s. The participants were instructed to pedal at a constant rate above 50 rpm for the duration of the test, which was stopped when the participants could not maintain this cadence and/or volitional exhaustion. Oxygen consumption during the exercise test was measured using a breath‐by‐breath metabolic analyser (Cortex Metalyzer 3B, Cortex Biophysik, Leipzig, Germany). V̇O_2max_ was calculated as the highest 30‐s average value, and peak power output was defined as the highest power achieved (measured in W).

#### 2.3.2 Blood Sampling Procedure

For each sample, 3 mL of venous blood was drawn from the antecubital vein of the seated participant. The blood was allowed to clot at room temperature and then centrifuged at 3500 g for 20 min to separate the serum. The serum was collected and stored at −20°C for subsequent analysis. The cTnT, testosterone and cortisol levels were measured quantitatively based on electrochemiluminescence technology using a Cobas E 411 analyser (Roche Diagnostics Ltd., Rotkreuz, Switzerland). The cTnT assay is a fifth‐generation high‐sensitivity immunoassay and has a lower detection limit of 3 ng.L^−1^ with an upper limit of 10,000 ng.L^−1^. The coefficient of variation for a mean cTnT concentration of 13.5 ng.L^−1^ is 5.2% (Giannitsis et al. 2010). The intra‐assay coefficients of variation for testosterone and cortisol are 4.7% and 12.7% for a mean at 0.095 ng.mL^−1^ and 3.09 nmol.L^−1^, respectively, whereas the limits of detection are 0.025 ng.mL^−1^ and 1.5 nmol.L^−1^, respectively. All assays were performed according to the manufacturer’s instructions.

#### 2.3.3 Physical Activity and Sedentary Behaviour Measurement

A triaxial accelerometer (ActiGraph wGT3x‐BT, ActiGraph Corporation) was used to measure physical activity patterns. The participants wore the accelerometer on their nondominant wrist for at least 10 h per day over two consecutive days. The accelerometer sampled data at 100 Hz, which were summed over 1‐min epochs and processed using the ActiLife software (version 6.13.0, ActiGraph Corporation). Daily physical activity and sedentary behaviour were calculated based on the minutes of recorded data. Sedentary time was defined as ≤ 100 counts.min^−1^, light physical activity as 101–1951 counts.min^−1^ and moderate to vigorous physical activity (MVPA) as ≥ 1952 counts.min^−1^ (Freedson et al. 1998).

### 2.4 Statistical Analysis

The data were first assessed for normality using the Kolmogorov–Smirnov test. If the data exhibited a skewed distribution, then log transformation was applied to achieve normality. A two‐way repeated measures analysis of variance (ANOVA) was used to evaluate differences in cTnT levels across the three trials (GXT1, CON and GXT2) and two time points (pre‐ and post‐exercise). A one‐way repeated measures ANOVA was used to analyse the differences in exercise duration, V̇O_2_, heart rate and RPE across the three trials. Mauchly’s test of sphericity was applied to assess the assumption of sphericity; if violated, then the Greenhouse–Geisser correction was applied. When significant main effects or interactions were observed, the Newman–Keuls test was used for post hoc comparisons. Partial eta squared (*ηp* ^2^) was used to measure interaction effects: 0.04 (small), 0.25 (medium) and 0.64 (large). Additionally, a paired Student’s *t*‐test was employed to compare peak power and V̇O_2max_ between GXT1 and GXT2. Statistical significance was set at *p* < 0.05. Data analysis was conducted using SPSS Statistics 20.0 (IBM Corp., Armonk, NY, USA).

## 3 Results

Twenty‐one participants completed the study without any reported adverse events during or after exercise. As expected, the acute exercise data for GXT1—such as exercise duration, peak power, V̇O_2max_, mean V̇O_2_, mean heart rate, maximum heart rate and RPE—were similar to those for GXT2 (all *p* > 0.05). As expected, mean V̇O_2_, mean heart rate, maximum heart rate and RPE were significantly lower (all *p* < 0.05) for CON compared with GXT1 and GXT2 (Table 1).

**TABLE 1:** Acute exercise or control data.

	Duration (min)	Power_peak_ (W)	V̇O_2max_ (mL.kg^−1^.min^−1^)	V̇O_2mean_ (mL.kg^−1^.min^−1^)	HR_mean_ (beat.min^−1^)	HR_max_ (beat.min^−1^)	RPE
GXT1	7.0 ± 1.4	236 ± 41	31.8 ± 8.7	20.7 ± 5.6	143 ± 11	184 ± 14	19 ± 1
CON	7.0 ± 1.4	—	—	4.2 ± 0.6*	78 ± 8*	94 ± 8*	7 ± 1*
GXT2	7.0 ± 1.4	236 ± 41	31.8 ± 6.4	20.9 ± 3.7	141 ± 13	182 ± 17	18 ± 2

*Note:* The data are presented as the mean ± standard deviation. A one‐way repeated measures analysis of variance was used to compare differences in exercise duration, mean V̇O_2_ (V̇O_2mean_), mean and max heart rate (HR_mean_ and HR_max_, respectively) and rating of perceived exertion (RPE) across the trials (GXT1, CON and GXT2). When there were significant main effects, the Newman–Keuls test was used for post hoc comparisons. A paired Student’s *t*‐test was employed to compare peak power output (Power_peak_) and V̇O_2max_ between GXT1 and GXT2. The asterisk (*) indicates a significant difference compared with GXT1 and GXT2 (*p* < 0.05).

The duration of sedentary behaviour, light physical activity and moderate‐to‐vigorous physical activity was consistent (all *p* > 0.05) across the three trials on both the trial day and the day before (Table 2). The morning levels of testosterone and cortisol were also similar (both *p* > 0.05) across the three trials (Table 3).

**TABLE 2:** Physical activity and sedentary behaviour variables (min).

	Trial day^a^			Day before trial day		
	GXT1	CON	GXT2	GXT1	CON	GXT2
Sedentary behaviour	125 ± 82	112 ± 55	134 ± 98	461 ± 118	464 ± 153	464 ± 162
Light physical activity	197 ± 83	182 ± 27	186 ± 51	474 ± 107	489 ± 93	468 ± 101
MVPA	97 ± 34	92 ± 33	98 ± 30	96 ± 49	112 ± 59	114 ± 63

*Note:* The data are presented as the mean ± standard deviation. A one‐way repeated measures analysis of variance was used to examine differences in physical activity (sedentary behaviour, light physical activity and moderate‐to‐vigorous physical activity [MVPA]) across the trials (GXT1, CON and GXT2). There were no significant differences across the trials (*p* > 0.05).

^a^ The data on physical activity on the trial day concluded when blood collection was completed, which was 4 h post‐exercise.

**TABLE 3:** Serum hormones before (9 a.m.) exercise or control.

	GXT1	CON	GXT2
Testosterone (ng.mL^−1^)	5.70 ± 1.61	5.73 ± 1.31	5.46 ± 1.23
Cortisol (nmol.L^−1^)	424 ± 197	466 ± 230	477 ± 170

*Note:* The data are presented as the mean ± standard deviation. A one‐way repeated measures analysis of variance was conducted to compare pre‐exercise hormone levels (testosterone and cortisol) across the three trials (GXT1, CON and GXT2). There were no significant differences in hormone levels between the trials (*p* > 0.05).

**FIGURE 2:**
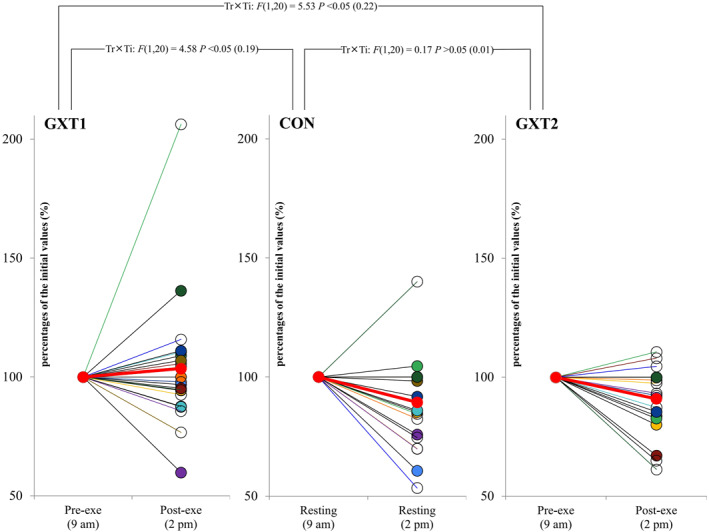
Cardiac troponin T levels in individuals (*n* = 21) across the three trials. Individual data points are represented by circles, with values for the same participant connected by lines. Each subject is assigned a unique colour for their individual line or circle across the three trials. The thick red line represents the average of all participants. All values were normalised to percentages of their initial values. **GXT1**, the first trial, a graded exercise test to determine V̇O_2max_; **CON**, the second trial, resting control; **GXT2**, the third trial, the same graded exercise test as GXT1. Measurements were taken before exercise (pre‐exe, 9 a.m.) and 4 h after exercise (post‐exe, 2 p.m.). Two‐way repeated measures analysis of variance interaction [trials (Tr) × time (Ti)]: F‐ratio, *p* value (ηρ^2^).

The cTnT levels for GXT1, CON and GXT2 are shown as cohort data in Table 4 and as individual percentage changes from their initial values in Figure 2. There was no significant change (*p* > 0.05) in the cTnT levels from pre‐ to post‐exercise for GXT1. The cTnT levels decreased significantly (*p* < 0.05) over time for both CON and GXT2. Moreover, there is a significant interaction for the cTnT levels between GXT1 and CON (*p* < 0.05, *ηp* ^2^ = 0.19) and between GXT1 and GXT2 (*p* < 0.05, *ηp* ^2^ = 0.22). However, there was no interaction for the cTnT levels between CON and GXT2 (*p* > 0.05, *ηp* ^2^ = 0.01).

**TABLE 4:** Serum cardiac troponin T [cTnT, ng.L^−1^] before (pre‐exe, 9 a.m.) and 4 h (post‐exe, 2 a.m.) after three trials.

	GXT1	CON	GXT2
Pre‐exe	3.80 (3.00–10.59)	3.52 (3.00–10.84)	4.13 (3.00–15.48)
Post‐exe	4.22 (3.00–9.08)	3.00 (3.00–7.57)*	3.24 (3.00–12.96)*

*Note:* The data are presented as the median (range). After log transformation, a two‐way repeated measures analysis of variance was performed to analyse changes in serum cTnT levels across the time points (pre‐exe and post‐exe) and trials (GXT1, CON and GXT2). When there were significant main effects, the Newman–Keuls test was used for post hoc comparisons. The asterisk (*) indicates a significant difference relative to the corresponding pre‐exe value (*p* < 0.05).

## 4 Discussion

### 4.1 Overview of the Findings

To the best of our knowledge, this is the first study to evaluate the circadian variance in cTn to help interpret exercise‐induced cTn after a GXT. The main findings of this study are as follows: (1) in young males, a GXT appears to prevent the natural circadian decline in cTnT across a daytime period and (2) we present possible evidence of late “exercise preconditioning” that is induced by an initial GXT 7−9 days before a second GXT.

### 4.2 Circadian Variation and GXT

After GXT1, there was no significant change in the cTnT levels from pre‐ to post‐exercise. This result is not surprising, considering the relatively low workload of a GXT compared with prolonged strenuous exercises such as marathons, which have been shown to lead to increased cTnT levels substantially (Aengevaeren et al. 2021). Although there are limited studies on cTn levels before and after GXT in healthy individuals, there is an abundance of research highlighting significant cTnT increases after a GXT in patients with cardiovascular disease (Samaha et al. 2019). Of note, only two studies have reported a significant increase in cTnI levels following GXT in healthy individuals; both involved longer exercise durations of around 41 min in trained athletes (Bjørkavoll‐Bergseth et al. 2021; Bjorkavoll‐Bergseth et al. 2024), which contrasts with the 7‐min duration of GXT in the present study. Given that our participants are young individuals who do not regularly engage in exercise, the 7‐min GXT duration appears reasonable (Beltz et al. 2016).

An analysis of the cTnT response for the CON trial offers further insights into the circadian variation of cTnT. Specifically, the cTnT levels were significantly reduced during CON, exhibiting a circadian decline from 9 a.m. to 2 p.m (Klinkenberg et al. 2014). Furthermore, the significant interaction between changes in the cTnT levels across CON and GXT1 suggests that the GXT1 response may mediate this circadian variation or reflect a biologically driven increase in cTnT when compared with the natural circadian decline. This result is partially supported by the findings presented by Klinkenberg et al. (2014), who observed that three 15‐min slow‐paced walking trials (800–1000 m) did not completely offset the circadian decrease in cTnT. Because exercise intensity, rather than duration, is considered to be a more significant trigger for cTn release (Fu et al. 2009), the variation in exercise intensity between studies could explain the different results.

Our findings suggest that the effect of a morning GXT on cTn levels may be underestimated due to the circadian fluctuation in cTn. Future studies should explore whether performing a GXT in the evening, when cTn levels naturally rise due to circadian rhythms (Klinkenberg et al. 2014), might lead to an overestimation of exercise‐induced cTn elevation. Gaining a better understanding of the cTn response to GXT at different times of day could provide valuable insights into its potential prognostic significance.

## 5 Sustainability of Preconditioning

In contrast to GXT1, cTnT levels decreased significantly following GXT2. Furthermore, there was an interaction between GXT1 and GXT2 regarding changes in cTnT levels, but there was no such interaction between GXT2 and CON. The interpretation of these differences is complex, but they likely reflect a component of late exercise preconditioning rather than an absence of cTnT release following GXT2. To our knowledge, this study is the first to demonstrate that when young participants perform two identical GXT bouts separated by 7–9 days, the post‐exercise trajectory of cTnT is notably altered after the second GXT. We observed a similar phenomenon in our previous research, where exercise bouts were separated by 4 or 48 h (Nie, George, Tong, Tian, et al. 2011; Zhang et al. 2019). These earlier studies suggested that exercise preconditioning at both early and late protection stages may reduce the cTnT response to subsequent exercise. The current study has added to these previous findings by revealing that late protective effects can persist for up to 7−9 days. These valuable human data support animal research demonstrating that the protective effects of a single or short‐term exercise exposure may last up to 9–11 days (Thijssen et al. 2022).

Interestingly, the findings from the present study contrast with our previous study, where we found that after prolonged moderate‐intensity exercise, cTnT levels remained comparable across eight consecutive rest days (Zhang et al. 2019). Given that preconditioning efficacy is reduced in females or those with cardiovascular risk factors such as obesity (Thijssen et al. 2018, 2022), we speculate that the overweight status of the female participants in our previous study (Zhang et al. 2019) may have impaired or shortened the period of late cardioprotection. To investigate this possibility, future research should incorporate sex differences and cardiovascular risk factors. Furthermore, given that different exercise protocols may have influenced the outcomes of the two studies, an important and more ecologically relevant question arises: how long do the effects of moderate‐intensity continuous exercise—an exercise protocol more commonly used in clinical settings—last on cTn levels after a subsequent GXT? Addressing this question is crucial as it has direct implications for optimising the scheduling of GXT.

### 5.1 Individual Variability in the cTnT Response

Our study corroborates previous research demonstrating considerable interindividual variation in the cTnT response to exercise (Tian et al. 2012). Our findings reinforce the idea that exercise‐induced cTn elevation is influenced by a range of interacting factors (Eijsvogels et al. 2015). We observed substantial variability among the participants in the cTnT response to exercise; however, this variation could not be fully explained by conventional factors such as the workload (internal or external); time of day; environment; daily physical activity; diet or participant characteristics such as age, body mass and cardiorespiratory fitness. Notably, the degree of interindividual variability differed across the three trials, with a coefficient of variation of 27% for GXT1, 21% for CON and 15% for GXT2. Thus, our study highlights the potential role of chronobiological influences and prior exercise exposure, factors that have not been previously considered to influence the interindividual variability of exercise‐induced cTnT levels (Eijsvogels et al. 2015).

A unique aspect of this study is the inclusion of a nonexercise control condition, allowing for the assessment of individual variability in cTnT levels in the absence of acute exercise. Interestingly, we observed considerable interindividual differences in cTnT levels even for the CON trial, suggesting that baseline physiological variation may contribute to differences in exercise‐induced cTnT responses. This finding underscores the need for further research to explore underlying mechanisms, including genetic predisposition, which may influence individual variability in cTnT kinetics.

### 5.2 Implications

The underlying mechanisms and clinical significance of the GXT‐related cTnT response, considering natural circadian variance, as well as the potential evidence of “late cardioprotection,” remain unclear and are beyond the scope of this study. Recent studies have shown that exercise‐induced increases in cTn are associated with elevated cardiovascular risk, challenging the assumption that post‐exercise cTn elevation is benign (Aengevaeren et al. 2019; Kleiven et al. 2020; Skadberg et al. 2017). Our findings provide new insights, warranting further investigation into the diagnostic or prognostic potential of post‐GXT cTn in young healthy males.

First, our study demonstrates that even a GXT with a very low workload can “elevate” cTnT levels in healthy young men within the context of natural circadian variance. This suggests that short, standardised post‐GXT cTn measurements could be a feasible and practical option for future clinical exploration. It is worth noting that for patients with cardiovascular disease, incorporating cTn data into a GXT could enhance the diagnostic utility compared with resting cTn measurements alone (Liebetrau et al. 2015). Our work provides the potential for applying this approach to young healthy males.

Second, the current study suggests that exercise preconditioning may last longer than previously expected, complicating the scheduling of tests for cTn observation post‐exercise. Future studies should take this into account when determining the timing of cTn measurements, as it has important implications for both research and clinical practice.

### 5.3 Strengths and Limitations

A key strength of this study is its robust design: we controlled potential confounding variables. Precise scheduling of the GXT and CON trials, coupled with the standardised lab settings, effectively minimised the influence of external workload and environmental factors on circadian variation and/or late exercise preconditioning. Additionally, physical activity patterns, as well as morning testosterone and cortisol levels (reflecting basal psychological and physiological stress), were consistent across all three trials. This consistency suggests minimal confounding effects on the results (Fabre‐Estremera et al. 2023; Hammadah et al. 2018), supporting the validity of our findings.

Despite these strengths, certain limitations must be acknowledged. To minimise confounding factors, we conducted the formal experimental trials within a short timeframe. However, this approach introduced a limitation: the order of GXT1 and CON was not randomised. Additionally, we only collected blood samples before exercise and 4 h post‐exercise to minimise participant burden, including reducing blood sampling trauma and participation duration. Consequently, the lack of serial post‐exercise cTnT measurements may result in missing peak values, although 3−4 h post‐GXT is the optimal time to capture the peak (Liebetrau et al. 2015). Finally, we focused on young males without an exercise background. Given that females or trained individuals exhibit different baseline cTn levels and responses to exercise (Kong et al. 2017; Nie, George, Tong, et al. 2011), future studies should include these groups to enhance diversity and improve the generalisability of the findings.

## 6 Conclusion

In conclusion, a GXT influences blood cTnT levels in young males by preventing the natural daytime circadian decline. Additionally, late exercise preconditioning, from an initial GXT, is potentially evident as cTnT levels decline after a second GXT 7−9 days after the first exercise trial. This study provides initial grounds for further research into the diagnostic or prognostic potential of post‐exercise cTnT change in the general population.

## Ethics Statement

All procedures performed in studies involving human participants were in accordance with the ethical standards of the institutional research committee and with the 1964 Helsinki Declaration and its later amendments or comparable ethical standards.

## Conflicts of Interest

The authors declare no conflicts of interest.
